# *Echinococcus multilocularis* and *Echinococcus shiquicus* in a small mammal community on the eastern Tibetan Plateau: host species composition, molecular prevalence, and epidemiological implications

**DOI:** 10.1186/s13071-018-2873-x

**Published:** 2018-05-16

**Authors:** Xu Wang, Jiayu Liu, Qingqiu Zuo, Zhiqiang Mu, Xiaodong Weng, Xiaohui Sun, Junyao Wang, Belgees Boufana, Philip S. Craig, Patrick Giraudoux, Francis Raoul, Zhenghuan Wang

**Affiliations:** 1 School of Life Sciences, East China Normal University, Shanghai, China; 2 Department of Infectious, Parasitic and Immuno-Mediated Diseases, Istituto Superiore di Sanità, Rome, Italy; 3 School of Environment and Life Sciences, University of Salford, Greater Manchester, UK; 4 Chrono-Environment Lab, University of Bourgogne-Franche-Comté and CNRS, Besançon, France

**Keywords:** *Echinococcus multilocularis*, *E. shiquicus*, Small mammal, Prevalence, Tibetan Plateau

## Abstract

**Background:**

The eastern part of the Tibetan Plateau is now recognized as an endemic region with the highest reported human infection rates in the world of human alveolar echinococcosis (AE) caused by *Echinococcus multilocularis*. Existing epidemiological studies on AE have mainly focused on the synanthropic environment, while basic parasitological and ecological aspects in wildlife host species remain largely unknown, especially for small mammal hosts. Therefore, we examined small mammal host species composition, occurrence, and the prevalence of both *E. multilocularis* and *E. shiquicus* in Shiqu County (Sichuan Province, China), eastern Tibetan Plateau.

**Results:**

In total, 346 small mammals from five rodent and one pika species were trapped from four randomly set 0.25 ha square plots. Two vole species, *Lasiopodomys fuscus* (*n* = 144) and *Microtus limnophilus* (*n* = 44), and the plateau pika (*Ochotona curzoniae*) (*n* = 135), were the three most-dominant species trapped. Although protoscoleces of *E. multilocularis* and *E. shiquicus* were only observed in *L. fuscus* and *O. curzoniae*, respectively, *cox*1 and *nad*1 gene DNA of *E. shiquicus* was detected in all the small mammal species except for *Neodon irene*, whereas *E. multilocularis* was detected in the three most-dominant species. The overall molecular prevalence of *Echinococcus* species was 5.8 (95% CI: 3.3–8.2%) ~ 10.7% (95% CI: 7.4–14.0%) (the conservative prevalence to the maximum prevalence with 95% CI in parentheses), whereas for *E. multilocularis* it was 4.3 (95% CI: 2.2–6.5%) ~ 6.7% (95% CI: 4.0–9.3%), and 1.5 (95% CI: 0.2–2.7%) ~ 4.1% (95% CI: 2.0–6.1%) for *E. shiquicus*. The prevalence of both *E. multilocularis* and *E. shiquicus*, was significantly higher in rodents (mainly voles) than in pikas. Phylogenetic analyses revealed that *Echinococcus* haplotypes of *cox*1 from small mammal hosts were actively involved in the sylvatic and anthropogenic transmission cycles of *E. multilocularis* in the eastern Tibetan Plateau.

**Conclusions:**

In contrast to previous studies, the current results indicated that rodent species, rather than pikas, are probably more important natural intermediate hosts of *E. multilocularis* and *E. shiquicus* in the eastern Tibetan Plateau. Thus, understanding interspecific dynamics between rodents and pikas is essential to studies of the echinococcosis transmission mechanism and human echinococcosis prevention in local communities.

**Electronic supplementary material:**

The online version of this article (10.1186/s13071-018-2873-x) contains supplementary material, which is available to authorized users.

## Background

Echinococcosis, caused by *Echinococcus* spp. tapeworms, is a severe zoonosis with a worldwide distribution. Among the ten recognized species [1], *Echinococcus granulosus* (*sensu stricto*) and *E. multilocularis* are the two most widely distributed species, causing human cystic echinococcosis (CE) and alveolar echinococcosis (AE), respectively [2]. Both CE and AE are significant public health problems in the pasture areas of China [3], especially AE on the eastern Tibetan Plateau, which is the most pathogenic form of echinococcosis, and lethal in the absence of treatment; 91% of new cases annually worldwide occurred in China [4]. The eastern part of the Tibetan Plateau is now recognized as an endemic region with the highest reported human infection rates in the world [5, 6]. Thus, echinococcosis has been listed as a critical endemic disease, and patients are eligible for free treatment from the national medical system in China [7].

As a typical example of the endemicity of *Echinococcus* spp. in the eastern Tibetan Plateau, the highest human echinococcosis infection rate in the world (12.9%) was detected in Shiqu County, Sichuan Province [5]. Three *Echinococcus* species, *E. granulosus* (*s.s.*), *E. multilocularis*, and *E. shiquicus*, coexist in this region [5, 6, 8, 9]. *Echinococcus granulosus* (*s.s.*) was confirmed to be mainly transmitting among synanthropic hosts, such as dogs and livestock, while transmission patterns of *E. multilocularis* and *E. shiquicus* involve complex sylvatic cycles that include several wildlife species [3]. The sylvatic transmission cycle of *E. multilocularis* in this area comprises the main definitive host species, *Vulpes ferrilata* (the Tibetan fox), and several intermediate host small mammals species (rodents and lagomorphs) [8, 10]. By preying on small wild mammals, dogs bring the parasite into a synanthropic transmission ecosystem [3, 11]. *Echinococcus shiquicus* was originally thought to be transmitted only between *V. ferrilata* and *Ochotona curzoniae* (the plateau pika) [12]. However, although no human cases have yet been reported, dogs were found to have *E. shiquicus* DNA in their feces, and a role for dogs in the transmission of *E. shiquicus* is unknown [13].

Nevertheless, existing epidemiological studies have mainly focused on human communities and their domestic animals (e.g. dogs). Parasitological and ecological studies on how echinococcosis is transmitted and maintained in wildlife host species are rare [3, 10, 14]. For example, ecological and parasitological information about *E. multilocularis* in *V. ferrilata* is lacking (but see [15]). The prey species of *V. ferrilata* comprise several small mammal intermediate host species, mainly *O. curzoniae* and vole species [16]. Reports of *E. multilocularis* prevalence in intermediate host species on the eastern Tibetan Plateau have mainly focused on *O. curzoniae* and *Lasiopodomys fuscus* (the Smokey vole) [8, 17, 18]. However, other small mammals such as *Phaiomys leucurus* (the Blyth’s mountain vole), *Microtus limnophilus* (the lacustrine vole), *Neodon irene* (the Irene’s mountain vole), and *Cricetulus kamensis* (the Kam dwarf hamster) can also be abundant locally and could contribute to transmission [19]. He et al. [20] and Zhao [21] reported infection rates of *E. multilocularis* in small mammal communities of western Sichuan and southern Qinghai Provinces, respectively, but without clear reports of sampling design and species identification, evaluation of the relative importance of each small mammal host species in transmission is difficult. The need to prevent and control echinococcosis in local communities on pastures on the Tibetan Plateau requires improved understanding of the transmission ecology of *Echinococcus* spp. in their wildlife reservoir hosts. In particular, there is a crucial need to understand the composition of small mammal host species and the prevalence of *E. multilocularis* and *E. shiquicus* in this region.

Therefore, we studied the occurrence and prevalence of *E. multilocularis* and *E. shiquicus* in the small mammal community in Shiqu County on the eastern Tibetan Plateau. The genetic diversity of *Echinococcus* isolates recovered from this region was analyzed. Based on our results, we discuss the potential contribution of each host species during the transmission of echinococcosis in the local area.

## Methods

### Study area

Field studies were conducted at Yunbo Gou (33°11'N, 97°39'E) in northwestern Shiqu County (Ganze Tibetan Autonomous Prefecture, Sichuan Province, China) with an elevation of 4200–4700 m above sea level. Habitat vegetation is primary *Kobresia* meadow, with shrubs, mainly *Potentilla fruticosa* and *Salix cupularis*, from the middle to the summit of some hills. Wang et al. [22] classified the vegetation in this region into four categories: grassland; grassland and shrub; shrub; and disturbed areas. Grassland was the main vegetation type, covering > 90% of the study area [23]. The warm season extends from late June to mid-August, and is the suitable time for small mammal capturing.

### Sampling of small mammals

Small mammals were collected between July and August 2014, during the annual wildlife plague (*Yersinia pestis*) surveillance, conducted by the Shiqu County Center for Disease Control (Shiqu CDC). Four 50 × 50 m trapping plots were randomly set on grassland at Yunbo Gou. Small mammals in the plot were trapped using break-back traps (size: 12 × 6.5 cm) set at the entrance of their dens. In total, 400 traps were set in each trapping plot. The trapping period of each plot was set for 24 h (10:00 h to 10:00 h the next day).

Each small mammal was sexed and its body measured; the head was stored in a 50 ml capped tube with 95% ethanol for further species identification in the laboratory. The bodies were then dissected, and any lesions of *Echinococcus* spp. in the thoracic and peritoneal cavities and organs were carefully checked. When a lesion was detected, a small portion was cut and checked under a microscope for presence of protoscoleces. To a typical *Echinococcus* lesion, protoscoleces can be checked out, while to those atypical lesions, ones that were either too small (e.g. with a diameter < 1 mm) or calcified, protoscoleces could not be observed. Therefore, to further confirm the existence of *Echinococcus* spp., samples of each typical and atypical lesion and from livers of small mammals without visible lesions were stored separately in 2 ml storage tubes in 95% ethanol and stored at -20 °C for further PCR analysis.

### Small mammal species identification

Small mammal species identification was based on pelt color patterns, body measurement data, and skull-mandible morphological characteristics (e.g. of the molars) according to Luo et al. [24] and Smith & Xie [25]. To further confirm our identification, the specimens were compared with small mammal species specimens of the museum of the Northwest Institute of Plateau Biology, Xining.

### DNA extraction and PCR

DNA extraction from samples (i.e. lesion and tissue samples) was conducted using the MiniBEST Universal Genomic DNA Extraction Kit Ver.5.0 (TaKaRa) according to the manufacturer’s instructions. To identify *Echinococcus* spp., we used four primer pairs to perform parallel PCR tests of each sample. A Taeniidae family universal primer pair CO1JP2 (F/COI and R/COI, Table 1) [26] was used to amplify a region of approximately 874 bp in length of the mitochondrial *cox*1 gene. Three species-specific *nad*1 gene primer pairs (ND1Eg, ND1Em and ND1Es) were used to detect *E. granulosus* (*s.s.*), *E. multilocularis* and *E. shiquicus*, respectively (Table 1) [27]. All PCRs were performed in 50 μl volumes with 4 μl template DNA, 1 μl of the primers (10 μmol/l), 1 μl of bovine serum albumin (BSA, TaKaRa, Dalian, China), and 25 μl Premix Taq (Ex Taq Version 2.0 plus dye, TaKaRa), made up to 50 μl with deionized H_2_O (dH_2_O). PCR of CO1JP2 comprised 30 cycles of 30 s at 94 °C, 45 s at 52 °C, 90 s at 72 °C, and a final extension step of 72 °C for 5 min. Parameters of the PCRs for the three *nad*1 species-specific primer pairs were: 94 °C for 5 min followed by 35 cycles of 94 °C for 30 s, 45 s at the annealing temperature of each primer pair (Table 1), 72 °C for 90 s, and then 72 °C for 10 min. All PCRs were run on a DNA thermal cycler (Bio-Rad, Hercules, CA, USA).

**Table 1:** Primer sequences, lengths of PCR amplicons and annealing temperatures

Primers	Original code	Species	Target genes	Primer sequences	Amplicon length (bp)	Annealing temperature (°C)	Reference
CO1JP2	COIF	Taeniidae gen. sp.	*cox*1	TTGAATTTGCCACGTTTGAATGC	875	52	[26]
	COIR			GAACCTAACGACATAACATAATGA			
ND1Em	EmF19/3	*E. multilocularis*	*nad*1	TAGTTGTTGATGAAGCTTGTTG	207	53	[27]
	EmR6/1			ATCAACCATGAAAACACATATACAAC			
ND1Es	EsF50	*E. shiquicus*	*nad*1	TTATTCTCAGTCTCGTAAGGGTCCG	442	60	[27]
	EsR73			CAATAACCAACTACATCAATAATT			
ND1Eg	Eg1F81	*E. granulosus*	*nad*1	GTTTTTGGCTGCCGCCAGAAC	226	62	[27]
	Eg1R83			AATTAATGGAAATAATAACAAACTTAATCAACAAT			

### Other samples for molecular analyses

DNA of *Echinococcus* spp. from other host species involved in local transmission cycles was also used in this study. These samples included: three *Echinococcus*-positive Tibetan fox fecal samples previously used by Jiang et al. [15]; four positive domestic dog fecal samples infected by *E. multilocularis* previously used by Boufana et al. [13]; and AE lesion samples from six human patients living in Shiqu County between 2002 and 2007 [28]. The pretreatment and copro-DNA extraction protocols of Tibetan fox and dog fecal samples followed Jiang et al. [15] and Boufana et al. [13], respectively. Treatment of the human AE samples followed the small mammal sample pretreatment and DNA extraction protocol as described above.

### PCR product cloning and sequencing

PCR products were subjected to agarose gel electrophoresis and stained with ethidium bromide (EB); positive screening results indicated that the target gene fragments had been amplified. Positive amplicons were excised carefully from the gel and purified with the TIAN gel Midi Purification Kit (TIANGEN, Beijing, China). Purified products were cloned into the T-Vector pMD 19 (TaKaRa) in strict accordance with product instructions and transformed into competent *Escherichia coli* cells. DNA sequencing was conducted by Shanghai Majorbio Bio-Pharm Technology Co. Ltd. The results were compared with the NCBI database. (http://www.ncbi.nlm.nih.gov/BLAST).

### Statistics

Percentages of each trapped mammal species were calculated and a Chi-square test was used to test the sex bias among them. Plateau pikas and vole species were main species of trapped mammals. Therefore, the different distribution patterns between voles and pikas were also tested using a Chi-square test.

Given that PCRs with different primer pairs might have different results, the prevalence of the same *Echinococcus* spp. detected by different primers could be inconsistent. Therefore, all the visually identified (i.e. individuals with typical and atypical lesions) and *Echinococcus* DNA-detected individuals were analyzed using a Chi-square test for trends in proportions to determine if the detection of the same *Echinococcus* spp. by different primers (i.e. *cox*1 and *nad*1) was significantly different. Meanwhile, we defined the conservative prevalence of *Echinococcus* spp. by the percentage of positive samples detected by both *cox*1 and *nad*1 primers, and the maximum prevalence by the percentage of positive samples detected using at least one of the two genes.

To study how body condition might influence the detection of echinococcosis, all the visually identified (i.e. individuals with typical and atypical lesions) and *Echinococcus* DNA-detected individuals were analyzed using logistic regression models with four variables: (i) relative body weight (RW): the body weight of each individual divided by the heaviest one of the same species collected in this study; (ii) relative head-body length (RHBL): the head-body length of each individual divided by the longest one of the same species collected in this study; (iii) cross effect of relative weight and head-body length (CWL), expressed using the product of (ii) and (iii) of each individual; and (iv) lesions: three types, (typical, atypical, and no obvious lesion). The generalized *R*^2^ [29] and AICc [30] of each model were calculated. The model with the lowest AICc was selected as the best model, while models with ΔAICc < 2 compared with the AICc of the best model were also selected.

The development of *Echinococcus* lesions is known to be positively related with age of the host [31–34]. Morris [35] recommended using the dry weight of eye lenses to evaluate age, as practiced by Burlet et al. [34, 36]. We could not use this method because no scrutinized age-eye lens weight analyses of our studied species have ever been reported in China. Thus, we used weight and body length to indicate the relative age of each trapped individual [35]. Logically, it should be easier to identify *Echinococcus* infection in larger, heavier and older individuals [35]. Given that body size can differ significantly among species, relative measurements (i.e. RW and RHBL) of each individual were calculated by dividing the measurement with the data of the largest or heaviest individual of its own species collected in this study.

All statistical analyses were conducted using R 3.4.0 (http://www.r-project.org).

### Phylogenetic analyses

To analyze the phylogenetic relationships between *E*. *multilocularis* collected from small mammal hosts and from other host species of both local and wider geographical transmission cycles, maximum likelihood trees (ML trees) and Bayesian inference trees based on *cox*1 gene fragment haplotypes were constructed. Haplotypes of *cox*1 sequences were acquired from this study and 18 *E. multilocularis cox*1 sequences were selected from GenBank. One *E. shiquicus* (from one of the three Tibetan fox fecal samples, F12033) and one *E. granulosus* (*s.s.*) *cox*1 sequences (GenBank accession ID: KJ628374.1) were used as outgroups (Additional file 1: Table S1). When selecting sequences online, only data from indigenous host species were used. We did not build trees based on *E. multilocularis nad*1 gene sequences because the *nad*1 amplicons in *E. multilocularis* were too short (Table 1) and online data from different geographical regions were insufficient. Phylogenetic trees based on *E. shiquicus* sequences were not given in this study because only limited molecular data from this species from only a small area of the eastern Tibetan Plateau are available on GenBank, which would result in trees of *E. shiquicus* being less informative than trees of *E. multilocularis*.

Before construction of the phylogenetic trees, sequences were edited (Bioedit 7.0.9 [37]) and aligned (ClustalX2 [38]). Haplotypes were summarized, and Tajima’s *D* and Fu’s *Fs* tests were conducted by DnaSP v.5 [39]. Substitution saturation of the sequence matrix was tested by DAMBE 5 [40]. jModelTest v.2.1.4 [41] was used to test for the best-fit models of nucleotide substitution. Finally, ML trees were constructed using MEGA 7 by setting a ‘GRT+I’ substitution model with 1000 bootstrap replications. Bayesian trees were constructed using MrBayes 3.2.4 [42] by setting the ‘TIM3+I’ substitution model, using Markov Chain Monte Carlo (MCMC) posterior probability estimation for 2,000,000-generation with a 1000-generation sampling interval, and discarding the first 25% aging samples when summing up each tree. The best Bayesian tree was then compiled and processed by FigTree 1.4.3 (http://tree.bio.ed.ac.uk/software/figtree). Finally, a network diagram was drawn using Network 5.0 (http://www.fluxus-engineering.com).

## Results

### Small mammal species composition

A total of 346 small mammals were captured from the four trapping plots in the study site in July and August 2014. Except for one, *Cricetulus longicaudatus* (long-tailed dwarf hamster), most small mammals trapped were pikas and voles: *L. fuscus* 41% (144/346); *O. curzoniae* 39% (135/346); *M. limnophilus* 13% (44/346); *P. leucurus* 5% (16/346); and *N. irene* 2% (6/346). No significant sex bias was detected from the trapped small mammal species (*χ*^2^ = 4.485, *df =* 5, *P* = 0.482). Pikas were only trapped in the first and second plots, whereas voles were mainly trapped from the third and fourth plots (Table 2). There were significant differences in the distribution of pikas and voles among the four trapping plots (*χ*^2^ = 267.660, *df* = 3, *P* < 0.001).

**Table 2:** Statistics of species, gender, and anatomy of captured rodents

Species (*n*)	No. captured in different quadrats (*n*)				Sex (*n*)			Lesions^a^
	No. 1	No. 2	No. 3	No. 4	Male	Female	Unknown	
*Cricetulus longicaudatus* Long-tailed dwarf hamster (*n* = 1)	1	–	–	–	–	1	–	–
*Phaiomys leucurus* Blyth's mountain vole (*n* = 16)	–	2	7	7	9	7	–	–
*Neodon irene* Irene's mountain vole (*n* = 6)	–	-	1	5	3	3	–	–
*Microtus limnophilus* Lacustrine vole (*n* = 44)	–	9	20	15	18	26	–	6
*Lasiopodomys fuscus* Smokey vole (*n* = 144)	–	14	43	87	74	68	2^b^	19 (4)
*Ochotona curzoniae* Plateau pika (*n* = 135)	65	70	–	–	58	77	–	37 (1)
Total (*n* = 346)	66	95	71	114	162	182	2	62

*Abbreviation*: *n* number of individuals

^a^The number of individuals with distinct pathological features/lesions (number of individuals with *Echinococcus* protoscoleces)

^b^Carcasses were partly damaged by raptors

### Prevalence of *Echinococcus* spp. in the small mammal community

Suspected *Echinococcus* lesions were found in 62 individuals. Lesions in the lungs were found in five *O. curzoniae* and in both liver and lungs in another five *O. curzoniae*. All the other lesions of the remaining 52 individuals were in the liver. Molecular analyses later detected *Echinococcus* mtDNA in 22 out of the 62 individuals, including all the 5 individuals with typical lesions (i.e. *E. multilocularis* in 4 *L. fuscus* and *E. shiquicus* in 1 *O. curzoniae*), in which *Echinococcus* protoscoleces were checked out (Additional file 1: Table S2). Among these 22 individuals, *E. multilocularis* lesions in 20 voles (i.e. 15 *L. fuscus* and 5 *M. limnophilus*) were in the liver, whereas in the two *O. curzoniae*, one had typical *E. shiquicus* lesions in both liver and lungs and the other one had atypical *E. multilocularis* lesions in the lungs. In other individuals without visible lesions, *E. shiquicus* mtDNA was detected in 13 individuals, while *E. multilocularis* was detected in 2 (see Additional file 1: Table S2 for details). Therefore, the overall maximum prevalence of *Echinococcus* in small mammals was 10.7% (37/346, 95% CI: 7.4–14.0%), and the conservative prevalence was 5.8% (20/346, 95% CI: 3.3–8.2%). The maximum prevalence of *E. multilocularis* was 6.7% (23/346, 95% CI: 4.0–9.3%) with a conservative prevalence of 4.3% (15/346, 95% CI: 2.2–6.5%), whereas the maximum prevalence of *E. shiquicus* was 4.1% (14/346, 95% CI: 2.0–6.1%) and the conservative prevalence was 1.5% (5/346, 95% CI: 0.2–2.7%) (Table 3). No *E. granulosus* (*s.s.*) infections were detected.

**Table 3:** Prevalence (%) of *Echinococcus* spp. in each small mammal species (no. of infected individuals detected/ total no. individuals, 95% confidence intervals as percentage)

Species	*E. multilocularis*		*E. shiquicus*		Overall *Echinococcus* spp.	
	Conservative prevalence	Maximum prevalence	Conservative prevalence	Maximum prevalence	Conservative prevalence	Maximum prevalence
*C. longicaudatus*	0 (0/1)	0 (0/1)	0 (0/1)	– (1/1)	0 (0/1)	– (1/1)
*P. leucurus*	0 (0/16)	0 (0/16)	0 (0/16)	6.3 (1/16; 0–18.1)	0 (0/16)	6.3 (1/16; 0–18.1)
*N. irene*	0 (0/6)	0 (0/6)	0 (0/6)	0 (0/6)	0 (0/6)	0 (0/6)
*M. limnophilus*	9.1 (4/44; 0–19.7)^a^	11.4 (5/44; 2.0–20.7)^c^	6.8 (3/44; 0–14.3)	11.4 (5/44; 2.0–20.7)^e^	15.9 (7/44; 5.1–26.7)^f^	22.7 (10/44; 10.3–35.1)^h^
*L. fuscus*	7.6 (11/144; 3.3–12.0)^b^	11.1 (16/144; 6.0–16.2)^d^	0.7 (1/144; 0–2.1)	1.4(2/144; 0–3.3)^e^	8.3 (12/144; 3.8–12.9)^g^	12.5 (18/144; 7.1–17.9)^i^
*O. curzoniae*	0 (0/135)^a,b^	1.5 (2/135; 0–3.5)^c,d^	0.7 (1/135; 0–2.2)	3.7 (5/135; 0.5–6.9)	0.7 (1/135; 0–2.2)^f,g^	5.2 (7/135; 1.4–8.9)^h,i^
Total	4.3 (15/346; 2.2–6.5)	6.7 (23/346; 4.0–9.3)	1.5 (5/346; 0.2–2.7)	4.1 (14 /346; 2.0–6.1)	5.8 (20/346; 3.3–8.2)	10.7 (37/346; 7.4–14.0)

Statistical results for *C. longicaudatus*, *P. leucurus* and *N. irene* are not provided due to small sample sizes

^a^Significant differences detected between *M. limnophilus* and *O. curzoniae* (*χ*^2^ = 8.737, *df =* 1, *P* = 0.003)

^b^Significant differences detected between *L. fuscus* and *O. curzoniae* (*χ*^2^ = 8.814, *df =* 1, *P* = 0.003)

^c^Significant differences detected between *M. limnophilus* and *O. curzoniae* (*χ*^2^ = 6.195, *df =* 1, *P* = 0.013)

^d^Significant differences detected between *L. fuscus* and *O. curzoniae* (*χ*^2^ = 9.169, *df =* 1, *P* = 0.002)

^e^Significant differences detected between *M. limnophilus* and *L. fuscus* (*χ*^2^ = 6.7785, *df =* 1, *P* = 0.009)

^f^Significant differences detected between *M. limnophilus* and *O. curzoniae* (*χ*^2^ = 14.506, *df =* 1, *P* < 0.001)

^g^Significant differences detected between *L. fuscus* and *O. curzoniae* (*χ*^2^ = 7.414, *df =* 1, *P* = 0.006)

^h^Significant differences detected between *M. limnophilus* and *O. curzoniae* (*χ*^2^ = 9.927, *df =* 1, *P* = 0.002)

^i^Significant differences detected between *L. fuscus* and *O. curzoniae* (*χ*^2^ = 3.718, *df =* 1, *P* = 0.05)

There was a significant difference between the use of *cox*1 *vs nad*1 in the detection of *Echinococcus* infection. For *E. shiquicus*, significantly more infections were detected using *nad*1 primers than with *cox*1 (*χ*^2^ = 10.480, *df* = 1, *P* = 0.001), whereas the use of *cox*1 primers detected more *E. multilocularis* infections than with *nad*1 (*χ*^2^ = 7.415, *df* = 1, *P* = 0.006).

There was a distinct pattern to the prevalence of *E. multilocularis* and *E. shiquicus* in each small mammal species. *Cricetulus longicaudatus* and *P. leucurus* were only detected with *E. shiquicus* DNA, whereas no *Echinococcus* infection was detected in *N. irene* (Table 4)*.* For the three most-dominant small mammal host species (*L. fuscus*, *O. curzoniae* and *M. limnophilus*), both *E. multilocularis* and *E. shiquicus* were detected. There were no mixed *Echinococcus* spp. infections or DNA detected in a single host individual (Table 3). Among the three most-dominant host species, the prevalence of *E. multilocularis* and *E. shiquicus* were significantly higher in *M. limnophilus*, whereas the prevalence of these two *Echinococcus* spp. in *O. curzoniae* was significantly lower. *Lasiopodomys fuscus* had an intermediate *Echinococcus* prevalence (Table 3). The maximum prevalence of *E. multilocularis* and the overall *Echinococcus* prevalence in *L. fuscus* were significantly higher than in *O. curzoniae* (Table 3). No significant prevalence bias was detected between male and female mammals, except for *M. limnophilus*, in which the maximum prevalence of *Echinococcus* was significantly higher in females than in males (detected individuals, female/male, 9/1; *χ*^2^ = 4.189, *df* = 1, *P* = 0.041).

**Table 4:** Variables of host body condition influencing the general maximum prevalence of *Echinococcus* species as revealed by the best logistic regression model

Log odds of significant variables ± SE				Model evaluation		
Relative body weight	Relative head-body length	Lesions^a^		AICc		Generalized *R*^2^ of the best model
		1 *vs* 0	2 *vs* 0	Best model	Null model	
-16.010 ± 4.558	19.134 ± 8.521	-21.982	-0.556	49.677	108.473	0.572

^a^Categorical data, no SE presented

*Abbreviations*: 0, individuals without visible lesions; 1, individuals with atypical lesions; 2, individuals with typical lesions; *SE* standard error

Regression model simulation revealed that *Echinococcus* infection was detected in individual hosts with significantly longer RHBL (log odds ratio, 19.134), while their RBWs were significantly lighter than those without infection (log odds, -16.010) (Table 4). Moreover, although typical lesions were useful signs of infection (with a log odds ratio of -0.556 relative to the molecular results), atypical lesions were misleading and caused significantly more misidentification of infections (log odds ratio of -21.982, Table 4).

### Phylogenetic relationships between *E. multilocularis cox*1 gene haplotypes

**Fig. 1:**
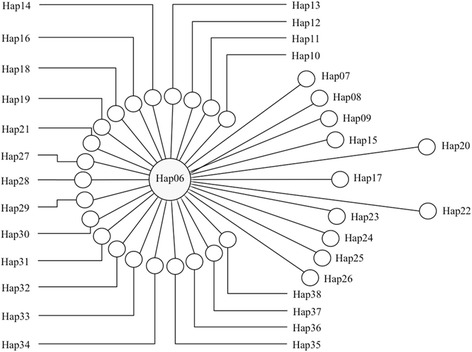
Network of 33 *Echinococcus multilocularis cox*1 gene haplotypes collected from samples in this study. The size of the circle represents the number of species of hosts with the *E. multilocularis* gene haplotype (Hap06 isolated from six species including humans, dogs, Tibetan foxes, two species of voles and plateau pikas, while each of the other haplotypes has only one host species, see Additional file 1: Table S1 for details). The distance between the circle centers shows the variation between two haplotypes (i.e. 1 bp mutation between Hap06 and Hap36)

In total, 45 haplotypes of a 768 bp long *E. multilocularis cox*1 gene fragment were acquired from 23 small mammals (76 sequences), six human AEs (6 sequences), four dog feces (4 sequences), and two Tibetan fox fecal samples (5 sequences) in this study, with an additional 17 sequences from online sources. Among the 76 sequences from Shiqu County, 33 haplotypes of *E. multilocularis* (i.e. Hap06-Hap38) were confirmed (Additional file 1: Table S1), of which Hap06 was the dominant haplotype, with 58 sequences covering all the human AE, dog, and Tibetan fox fecal samples, and 21 small mammal samples from *L. fuscus*, *M. limnophilus* and *O. curzoniae* (Fig. 1, Additional file 1: Table S1). The haplotype and nucleotide diversities for the *E. multilocularis cox*1 gene from the small mammal community in this study were 0.655 ± 0.064 and 0.0014 ± 0.0002, respectively. Significant negative values in both Tajima’s *D* (*D* = -2.83311, *P* < 0.001) and Fu’s *Fs* (*Fs* = -46.942, *P* < 0.001) tests indicated that the *E. multilocularis* population in the small mammal community of Shiqu County is currently expanding, and the dominant Hap06 haplotype may be under strong purifying selection.

**Fig. 2:**
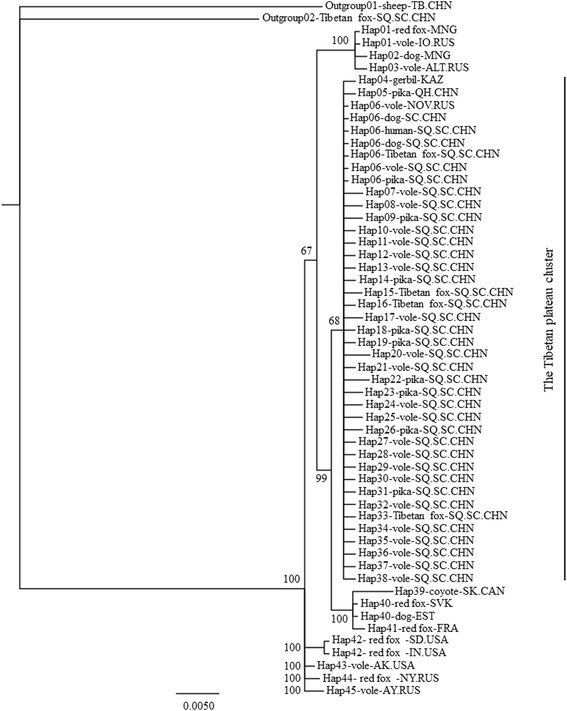
Phylogenetic tree comparing the geographical distribution between mtDNA *cox*1 gene haplotypes of *Echinococcus multilocularis*. The Bayesian phylogenetic analysis was used by setting the “TIM3+I” substitution model, 2,000,000-generation MCMC posterior probability estimation with a 1000-generation sampling interval, and discarding the first 25% samples when summing up trees

Both ML and Bayesian trees were constructed based on 56 sequences, including 54 *E. multilocularis* sequences and two *E. granulosus* (*s.s.*) and *E. shiquicus* sequences as outgroups (Additional file 1: Table S1). Both trees demonstrated identical topological relationships between haplotypes, but only the Bayesian tree is included here (Fig. 2) because of its more concise structure. The Tibetan Plateau cluster was distinctive from other geographical regions of the world, and mainly comprised the Shiqu haplotypes Hap06-Hap38, the Qinghai Province haplotype Hap05 (also from the Tibetan Plateau), and two other sequences (Hap04 and Hap06-Vole-NO.RUS, Additional file 1: Table S1) from neighboring geographical regions (Fig. 2).

## Discussion

Transmission of *E. multilocularis* and *E. shiquicus* relies on small mammal species as intermediate hosts [3]. Although monographs of the comprehensive taxonomy of small mammal species with coarse resolution distribution maps have been published [25, 43, 44], small mammal assemblages in western China still pose great challenges. Knowledge of the distribution, ecology, and even the basic taxonomy of these small mammals, especially rodent species, is lacking. Consequently, epidemiological research focused on small mammal host species based on basic taxonomic and ecological methodologies in specific *E. multilocularis*-endemic areas of China is limited [8, 10, 14]. Thus, our understanding of the transmission ecology of echinococcosis in the Tibetan Plateau ecosystem is far from complete.

### Small mammal host species community composition

Although all the six small mammal species reported in this study have been recorded previously on the Tibetan Plateau, information about small rodent species such as arvicolids and cricetids was largely lacking from Shiqu before the 21st century [45, 46]. He et al. [20] reported alveolar echinococcosis in the lagomorphs *Ochotona curzoniae* and *Lepus oiostolus*, and also in the commensal rodents *N. irene*, and *Mus musculus* in Shiqu. Raoul et al. [19] studied the habitat ecology of small mammal communities in Shiqu, reporting for the first time *P. leucurus* and *C. kamensis* in Sichuan Province. However, neither of these two studies reported *L. fuscus* in Shiqu. The distribution area of *L. fuscus* was judged to be restricted to southern Qinghai Province, and this was not considered to be a species distributed to Sichuan [24, 25, 43, 45]. However, in local plague surveillance studies [47, 48] and pest control schemes [49, 50], *L. fuscus* was judged to be a dominant rodent species in the high plateau pasture areas of northwest Sichuan. *Lasiopodomys fuscus* is morphologically similar to other vole species, such as *P. leucurus*, and thus could be included in reports in error if an accurate morphological identification is lacking [51]. Shiqu is located at the northwestern point of Sichuan Province, on the southern border of Qinghai; therefore, it is possible that what is thought to be the southern limit of the range of *L. fuscus* is incorrect [46]. The current study confirmed the presence of *L. fuscus* in Shiqu County, where it was the most abundant species of the five trapped rodent species and is likely to have a larger population than that of *O. curzoniae* in this region (Table 2).

### Importance of rodent species in the transmission of echinococcosis

Although the potential importance of rodent species as intermediate hosts of *E. multilocularis* on the eastern Tibetan Plateau has been mentioned previously elsewhere [10, 14, 51, 52], *O. curzoniae* were the most frequently examined small mammal host species with large sample sizes [8, 17, 18, 20, 21, 52–54]. For example, when evaluating the prevalence of *E. multilocularis*, Zhao [21] reported a prevalence of 15.2% in *O. curzoniae* (34/224) and 20% in *L. fuscus* (1/5 individuals). Similarly, Zhang & Wang [18] reported a prevalence of 5.3% in *O. curzoniae* (62/1177), but only 0.4% in *L. fuscus* (1/269). Thus, much smaller sampling sizes might be an important reason for the reported low prevalence of *E. multilocularis* in rodent species. In terms of *E. shiquicus*, *O. curzoniae* was previously the only confirmed intermediate host species [12, 54]. By contrast, our data revealed that, among the three most-abundant small mammal species, both *M. limnophilus* and *L. fuscus* had a significantly higher molecular prevalence of *E. multilocularis* and *E. shiquicus* than did *O. curzoniae* (Table 3). Moreover, *E. shiquicus* DNA was detected not only in the three most-abundant small mammal species, but also in the other two rodent species sampled (*P. leucurus* and *C. longicaudatus*) (Table 3). Although trapping data might not reflect the exact abundance of each species, the higher molecular prevalence of both *E. multilocularis* and *E. shiquicus* (Table 2) in rodents than in pikas suggests that rodent species are probably more important intermediate host species than pikas during the transmission of both *E. multilocularis* and *E. shiquicus*.

Traditionally, epidemiological studies of echinococcosis in western China mainly focused on human and domestic animal populations because of their obvious direct interactions especially regarding transmission of human CE and a role for dogs in risk of human AE. Phylogenetic analyses revealed that all 33 *cox*1 gene haplotypes from Shiqu County were closely related (Fig. 1), and clustered with haplotypes from other studies to form the Tibetan Plateau group, which is distinct from haplotypes from other geographical regions (Fig. 2). Hap06 was the dominant haplotype of *E. multilocularis* discovered in humans, domestic dogs, and almost all the wildlife host species tested in this study, including the three most dominant small mammal species (i.e. *O. curzoniae*, *M .limnophilus* and *L. fuscus*) (Fig. 2, Additional file 1: Table S1). These results confirm that small mammal species and dogs together comprise a wildlife and peri-domestic ecosystem for transmission of *E. multilocularis*. Thus, understanding the epidemiology in wildlife host species is pivotal to understanding the life-cycles and transmission ecology of these parasites.

For a parasite species such as *E. multilocularis*, which can utilize multiple host species, understanding the interspecific dynamics between host species is essential for understanding its transmission [55]. Both Tajima’s *D* and Fu’s *Fs* tests revealed the population expansion of *E. multilocularis* in the small mammal community in the study region, as supported by Nakao et al. [28]. Could this expansion be the result of the frequently reported increases in small mammal populations and their interspecific dynamics in western China? *Ochotona curzoniae* and several vole species are the main prey of the Tibetan fox (*V. ferrilata*) on grasslands of the eastern Tibetan Plateau [16]. The significant differences in prevalence of *E. multilocularis* among voles and *O. curzoniae* (Table 3) suggest that interspecific dynamics among these small mammal species could be essential factors influencing the predator-prey food chain, which would affect the epidemiology of *E. multilocularis* across all pasture areas on the Tibetan Plateau. For example, Wang et al. [56] reported a high density of both *O. curzoniae* and voles in open grassland areas within 2 km of villages. *Ochotona curzoniae* is usually blamed for degrading the grassland ecosystem of the eastern Tibetan Plateau and, consequently, has been a main target for poisoning to protect the grasslands [49, 50, 57]. Areas around villages are usually where poisoning programs are located. The fact that there was a significantly higher prevalence of *E. multilocularis* in vole species compared with *O. curzoniae* (Table 3) suggests that pika may have a lesser role compared to voles in the transmission of *E. multilocularis*. Furthermore the deliberate poisoning of *O. curzoniae* could result in increased densities of vole species, accompanied by a higher prevalence of *E. multilocularis* in wildlife, dogs and humans in local areas.

### Small mammal body condition and detection of *Echinococcus* DNA

The regression model revealed that individuals with larger RHBL were more likely to be detected with *Echinococcus*, based on the molecular tests (Table 4). Linear body dimensions are more or less correlated with age, especially in animals with short lifespans [35]. Thus, the results of the current study showed that older small mammals were more likely to have *E. multilocularis* or *E. shiquicus* infections. Similarly, Burlet et al. [34] reported that older *Arvicola terrestris* had a higher prevalence of *E. multilocularis* than younger animals. In small mammal hosts, *E. multilocularis* requires several months to grow from an oncosphere to a fertile metacestode [58]. Consequently, the development of metacestode lesions can be synchronized with the aging process of its hosts, such as rodents and pikas, further confirming the importance of understanding the host population structure and dynamics when evaluating the present infection burden and predicting the future trend of a specific parasitic species.

Although typical lesions were indicative of infection with specific *Echinococcus* species, as revealed by the regression model (Table 4), they were harder to find than were the frequently detected atypical lesions (Additional file 1: Table S2). However, low values of atypical lesions for determining *Echinococcus* infection (Table 4, Additional file 1: Table S2) required the use of molecular analyses. Nevertheless, such analyses provide the molecular-positive rate of the parasite, which is not equivalent to its true prevalence. For example, *E. shiquicus* DNA was detected in several rodent species; however, if no typical lesions with metacestodes were observed (Additional file 1: Table S2), then it was not possible to conclude that there was an established parasitic infection. Thus, the function of rodent species as natural intermediate hosts of *E. shiquicus* still requires further study. Moreover, many primers have been designed to test various nuclear [59, 60] and mitochondrial [15, 26, 27, 61] genes of *Echinococcus* species. In this study, the *cox*1 and *nad*1 genes were used. However, the significantly inconsistent results achieved with both genes (Additional file 1: Table S2) suggested that multiple parasite genes should be tested in the same epidemiological study. In addition, a prevalence interval that is based on the different levels of detection as determined by the available genes (e.g. the maximum and conservative prevalence defined in this study) would be more objective than using a single prevalence.

## Conclusions

Our observations suggest that rodent (vole) species are probably more important natural intermediate hosts of both *E. multilocularis* and *E. shiquicus* in Shiqu County on the eastern Tibetan Plateau. In addition to *O. curzoniae*, the small mammal community sustains echinococcosis transmission in the Tibetan ecosystem. Moreover, small mammal communities usually have complex intra- and interspecific relationships, which influence the population and spatial dynamics of each host species and, thus, the transmission patterns of alveolar echinococcosis and *E. shiquicus* in local areas. Consequently, we recommend that future studies on the epidemiology of human AE must consider the basic transmission ecology of the small mammal community as an essential component of research and for control purposes.

## Additional file

Additional file 1: **Table S1.** Information for *cox*1 sequences used in the Bayesian phylogenic tree in this study. **Table S2.** *Echinococcus* detection results of each suspected small mammal sample based on necropsy and molecular analyses. (DOCX 35 kb)

## Abbreviations

AE: alveolar echinococcosis
CDC: center for disease control
CE: cystic echinococcosis
CWL: cross effect of relative weight and head-body length
EB: ethidium bromide
ML: tree maximum likelihood tree
RHBL: relative head-body length
RW: relative body weight
*s.s.*: *sensu stricto*
