# Supplementary material for: Echinococcus multilocularis and Echinococcus shiquicus in a small mammal community on the eastern Tibetan Plateau: host species composition, molecular prevalence, and epidemiological implications
Source: Parasit Vectors. 2018 May 16;11:302. doi: 10.1186/s13071-018-2873-x (PMC5956848; doi:10.1186/s13071-018-2873-x)
Supplement: Supplementary file 1 — Table S1. Information for cox1 sequences used in the Bayesian phylogenic tree in this study. Table S2. Echinococcus detection results of each suspected small mammal sample based on necropsy and molecular analyses. (DOCX 35 kb) [file 13071_2018_2873_MOESM1_ESM.docx]

# Additional file 1: Table S1. Information for *cox*1 sequences used in the Bayesian phylogenic tree in this study

| **Haplotypes** | **Code in this study** | **NCBI accession number** | **Length (bp)** | **Species^#^** | **Host** | **Geographic Region** | **Reference** |
| --- | --- | --- | --- | --- | --- | --- | --- |
| Hap01 | Hap01-red fox-MNG | AB813186.1 | 1608 | *E.m.* | Red fox (*Vulpes vuipes*) | Mongolia (MNG) | NCBI |
|  | Hap01-vole-IO.RUS | AB777921.1 | 1608 | *E.m.* | Lake Baikal vole (*Alticola olchonensis*) | Irkutsk Oblast, Russia (IO.RUS) | [2] |
| Hap02 | Hap02-dog-MNG | AB813188.1 | 1608 | *E.m.* | Dog (*Canis lupus familiaris*) | Mongolia (MNG) | NCBI |
| Hap03 | Hap03-vole-ALT.RUS | AB777920.1 | 1608 | *E.m.* | Flat-headed vole (*A. strelzowi*) | Altai Republic, Russia (ALT.RUS) | [2] |
| Hap04 | Hap04-gerbil-KAZ | AB461415.1 | 1608 | *E.m.* | Gerbil (species name unknown) | Kazakhstan ( KAZ) | [3] |
| Hap05 | Hap05-pika-QH.CHN | AB491459.1 | 789 | *E.m.* | Plateau pika (*Ochotona curzoniae*) | Qinghai, China (QH.CHN) | [1] |
| Hap06 | Hap06-vole-NOV.RUS | AB688125.1 | 1608 | *E.m.* | Narrow-headed vole (*Microtus gregalis*) | Novosibirsk, Russia ( NOV.RUS) | [2] |
|  | Hap06-dog-SC.CHN | AB461417.1 | 1608 | *E.m.* | Dog (*Canis lupus familiaris*) | Sichuan, China (SC.CHN) | [3] |
|  | Hap06-human-SQ.SC.CHN | KY354093 | 830 | *E.m.* | Human (*Homo sapiens*), 6 sequences from 6 tissue samples | Shiqu, Sichuan, China (SQ.SC.CHN) | This study. |
|  | Hap06-dog-SQ.SC.CHN | KY354088 | 811 | *E.m.* | Dog (*C. lupus familiaris*), 4 sequences from 4 fecal samples | Shiqu, Sichuan, China (SQ.SC.CHN) | This study. |
|  | Hap06-Tibetan fox-SQ.SC.CHN | KY354083 | 875 | *E.m.* | Tibetan fox (*V. ferrilata*), 2 sequences from 2 fecal samples | Shiqu, Sichuan, China (SQ.SC.CHN) | This study. |
|  | Hap06-vole-SQ.SC.CHN | KY446474  KY446475 | 875  875 | *E.m.*  *E.m.* | Smokey voles (*Lasiopodomys fuscus*), 28 sequences  Lacustrine voles3 (*M. limnophilus*), 18 sequences | Shiqu, Sichuan, China (SQ.SC.CHN) | This study. |
|  | Hap06-pika-SQ.SC.CHN | KY446476 | 875 | *E.m.* | Plateau pikas (*O. curzoniae*), 3 sequences | Shiqu, Sichuan, China (SQ.SC.CHN) | This study. |
| Hap07 | Hap07-vole-SQ.SC.CHN | KY446506 | 875 | *E.m.* | Smokey vole (*L. fuscus*), 1 sequence | Shiqu, Sichuan, China (SQ.SC.CHN) | This study. |
| Hap08 | Hap08-vole-SQ.SC.CHN | KY446486 | 875 | *E.m.* | Lacustrine vole (*M. limnophilus*), 1 sequence | Shiqu, Sichuan, China (SQ.SC.CHN) | This study. |
| Hap09 | Hap09-pika-SQ.SC.CHN | KY446485 | 875 | *E.m.* | Plateau pika (*O. curzoniae*), 1 sequence | Shiqu, Sichuan, China (SQ.SC.CHN) | This study. |
| Hap10 | Hap10-vole-SQ.SC.CHN | KY446482 | 875 | *E.m.* | Smokey vole (*L. fuscus*), 1 sequence | Shiqu, Sichuan, China (SQ.SC.CHN) | This study. |
| Hap11 | Hap11-vole-SQ.SC.CHN | KY446484 | 875 | *E.m.* | Smokey vole (*L. fuscus*), 1 sequence | Shiqu, Sichuan, China (SQ.SC.CHN) | This study. |
| Hap12 | Hap12-vole-SQ.SC.CHN | KY446480 | 875 | *E.m.* | Smokey vole (*L. fuscus*), 1 sequence | Shiqu, Sichuan, China (SQ.SC.CHN) | This study. |
| Hap13 | Hap13-vole-SQ.SC.CHN | KY446497 | 875 | *E.m.* | Lacustrine vole (*M. limnophilus*), 1 sequence | Shiqu, Sichuan, China (SQ.SC.CHN) | This study. |
| Hap14 | Hap14-pika-SQ.SC.CHN | KY446487 | 875 | *E.m.* | Plateau pika (*O. curzoniae*), 1 sequence | Shiqu, Sichuan, China (SQ.SC.CHN) | This study. |
| Hap15 | Hap15-Tibetan fox-SQ.SC.CHN | KY354086 | 875 | *E.m.* | Tibetan fox (*V. ferrilata*), 1 sequence | Shiqu, Sichuan, China (SQ.SC.CHN) | This study. |
| Hap16 | Hap16-Tibetan fox-SQ.SC.CHN | KY354085 | 875 | *E.m.* | Tibetan fox (*V. ferrilata*), 1 sequence | Shiqu, Sichuan, China (SQ.SC.CHN) | This study. |
| Hap17 | Hap17-vole-SQ.SC.CHN | KY446478 | 875 | *E.m.* | Lacustrine vole (*M. limnophilus*), 1 sequence | Shiqu, Sichuan, China (SQ.SC.CHN) | This study. |
| Hap18 | Hap18-pika-SQ.SC.CHN | KY446479 | 875 | *E.m.* | Plateau pika (*O. curzoniae* ), 1 sequence | Shiqu, Sichuan, China (SQ.SC.CHN) | This study. |
| Hap19 | Hap19-pika-SQ.SC.CHN | KY446490 | 875 | *E.m.* | Plateau pika (*O. curzoniae*), 1 sequence | Shiqu, Sichuan, China (SQ.SC.CHN) | This study. |
| Hap20 | Hap20-vole-SQ.SC.CHN | KY446488 | 875 | *E.m.* | Lacustrine vole (*M. limnophilus*), 1 sequence | Shiqu, Sichuan, China (SQ.SC.CHN) | This study. |
| Hap21 | Hap21-vole-SQ.SC.CHN | KY446489 | 875 | *E.m.* | Smokey vole (*L. fuscus*), 1 sequence | Shiqu, Sichuan, China (SQ.SC.CHN) | This study. |
| Hap22 | Hap22-pika-SQ.SC.CHN | KY446481 | 875 | *E.m.* | Plateau pika (*O. curzoniae*), 1 sequence | Shiqu, Sichuan, China (SQ.SC.CHN) | This study. |
| Hap23 | Hap23-pika-SQ.SC.CHN | KY446495 | 875 | *E.m.* | Plateau pika (*O. curzoniae*), 1 sequence | Shiqu, Sichuan, China (SQ.SC.CHN) | This study. |
| Hap24 | Hap24-vole-SQ.SC.CHN | KY446503 | 875 | *E.m.* | Lacustrine vole (*M. limnophilus*), 1 sequence | Shiqu, Sichuan, China (SQ.SC.CHN) | This study. |
| Hap25 | Hap25-vole-SQ.SC.CHN | KY446504 | 875 | *E.m.* | Lacustrine vole (*M. limnophilus*), 1 sequence | Shiqu, Sichuan, China (SQ.SC.CHN) | This study. |
| Hap26 | Hap26-pika-SQ.SC.CHN | KY446505 | 875 | *E.m.* | Plateau pika (*O. curzoniae*), 1 sequence | Shiqu, Sichuan, China (SQ.SC.CHN) | This study. |
| Hap27 | Hap27-vole-SQ.SC.CHN | KY446500 | 875 | *E.m.* | Lacustrine vole (*M. limnophilus*), 1 sequence | Shiqu, Sichuan, China (SQ.SC.CHN) | This study. |
| Hap28 | Hap28-vole-SQ.SC.CHN | KY446491 | 875 | *E.m.* | Smokey vole (*L. fuscus*), 1 sequence | Shiqu, Sichuan, China (SQ.SC.CHN) | This study. |
| Hap29 | Hap29-vole-SQ.SC.CHN | KY446498 | 875 | *E.m.* | Lacustrine vole (*M. limnophilus*), 1 sequence | Shiqu, Sichuan, China (SQ.SC.CHN) | This study. |
| Hap30 | Hap30-vole-SQ.SC.CHN | KY446496 | 875 | *E.m.* | Lacustrine vole (*M. limnophilus*), 1 sequence | Shiqu, Sichuan, China (SQ.SC.CHN) | This study. |
| Hap31 | Hap31-pika-SQ.SC.CHN | KY446501 | 875 | *E.m.* | Plateau pika (*O. curzoniae*), 1 sequence | Shiqu, Sichuan, China (SQ.SC.CHN) | This study. |
| Hap32 | Hap32-vole-SQ.SC.CHN | KY446502 | 875 | *E.m.* | Lacustrine vole (*M. limnophilus*), 1 sequence | Shiqu, Sichuan, China (SQ.SC.CHN) | This study. |
| Hap33 | Hap33-Tibetan fox-SQ.SC.CHN | KY354087 | 872 | *E.m.* | Tibetan fox (*V. ferrilata*), 1 sequence | Shiqu, Sichuan, China (SQ.SC.CHN) | This study. |
| Hap34 | Hap34-vole-SQ.SC.CHN | KY446493 | 875 | *E.m.* | Smokey vole (*L. fuscus*), 1 sequence | Shiqu, Sichuan, China (SQ.SC.CHN) | This study. |
| Hap35 | Hap35-vole-SQ.SC.CHN | KY446499 | 875 | *E.m.* | Lacustrine vole (*M. limnophilus*), 1 sequence | Shiqu, Sichuan, China (SQ.SC.CHN) | This study. |
| Hap36 | Hap36-vole-SQ.SC.CHN | KY446507 | 875 | *E.m.* | Smokey vole (*L. fuscus*), 1 sequence | Shiqu, Sichuan, China (SQ.SC.CHN) | This study. |
| Hap37 | Hap37-vole-SQ.SC.CHN | KY446492 | 875 | *E.m.* | Lacustrine vole (*M. limnophilus*), 1 sequence | Shiqu, Sichuan, China (SQ.SC.CHN) | This study. |
| Hap38 | Hap38-vole-SQ.SC.CHN | KY446494 | 875 | *E.m.* | Smokey vole (*L. fuscus*), 1 sequence | Shiqu, Sichuan, China (SQ.SC.CHN) | This study. |
| Hap39 | Hap39-coyote-SK.CAN | KC550007.1 | 1605 | *E.m.* | Coyotes (*C. latrans*) | Saskatchewan, Canada (SK.CAN) | [4] |
| Hap40 | Hap40-red fox-SVK | AB461414.1 | 1608 | *E.m.* | Red fox (*V. vuipes*) | Slovakia (SVK) | [3] |
|  | Hap40-dog-EST | KT001423.1 | 1608 | *E.m.* | Dog (*C. lupus familiaris*) | Estonia (EST) | [5] |
| Hap41 | Hap41-red fox-FRA | AB461413.1 | 1608 | *E.m.* | Red fox (*V. vuipes*) | France (FRA) | [3] |
| Hap42 | Hap42- red fox -SD.USA | AB374425.1 | 1608 | *E.m.* | Red fox (*V. vuipes*) | South Dakota, US ( SD.US) | [6] |
|  | Hap42- red fox -IN.USA | AB461419.1 | 1608 | *E.m.* | Red fox (*V. vuipes*) | Indiana, America (IN.USA) | [3] |
| Hap43 | Hap43-vole-AK.USA | AB461418.1 | 1608 | *E.m.* | Vole (species name unknown) | Alask, America (AK.USA) | [3] |
| Hap44 | Hap44- red fox -NY.RUS | AB777916.1 | 1608 | *E.m.* | Arctic fox (*V. lagopus*) | Nizhnekolymsky, Yakutia,  Russia (NY.RUS) | [2] |
| Hap45 | Hap45-vole-AY.RUS | AB777918.1 | 1608 | *E.m.* | Narrow-headed vole (*M. gregalis*) | Anabarsky,Yakutia, Russia (AY.RUS) | [2] |
| Outgroup01 | Outgroup01-sheep-TB.CHN | KJ628374.1 | 1609 | *E.g.* | Sheep (*Ovis aries*) | Tibet, China (TB.CHN) | NCBI |
| Outgroup02 | Outgroup02-Tibetan fox-SQ.SC.CHN | KY328700 | 875 | *E.s.* | Tibetan fox (*V. ferrilata*) | Shiqu, Sichuan, China (SQ.SC.CHN) | This study. |

# *E.m.*, *Echinococcus multilocularis*; *E.s.*, *E. shiquicus*; *E.g.*, *E. granulosus.*

**Additional file 1:** **Table S2. *Echinococcus* detection results of each suspected small mammal sample based on necropsy and molecular analyses.**

|  | **Code** | **Plot no.** | **Species** | **Gender** | **Organs with**  **visible lesions** | **Lesions^b^** | **Body measurements** | | ***E.multilocularis*** | | ***E.shiquicus*** | | ***E.granulosus*** | |
| --- | --- | --- | --- | --- | --- | --- | --- | --- | --- | --- | --- | --- | --- | --- |
|  |  |  |  |  |  |  | **Weight**  **(g)** | **Body lenghth (mm)** | **COX1** | **ND1** | **COX1** | **ND1** | **COX1** | **ND1** |
| Lesion samples^a^ | HS14091 | 2 | *L.fuscus* | ♂ | liver | 1 | 65.9 | 126.8 | + | + | - | - | - | - |
|  | HS14153 | 3 | *L.fuscus* | ♂ | liver | 1 | 38.3 | 107.7 | + | + | - | - | - | - |
|  | HS14167 | 3 | *L.fuscus* | ♂ | liver | 2 | 34.4 | 99.1 | + | - | - | - | - | - |
|  | HS14170 | 3 | *L.fuscus* | ♂ | liver | 1 | 33.8 | 98.9 | + | + | - | - | - | - |
|  | HS14179 | 3 | *L.fuscus* | ♂ | liver | 1 | 35.9 | 105 | + | + | - | - | - | - |
|  | HS14187 | 3 | *L.fuscus* | ♂ | liver | 1 | 29.1 | 94.5 | + | - | - | - | - | - |
|  | HS14191 | 3 | *L.fuscus* | ♂ | liver | 1 | 22.4 | 85.4 | + | + | - | - | - | - |
|  | HS14194 | 3 | *L.fuscus* | ♂ | liver | 2 | 44 | 110 | + | + | - | - | - | - |
|  | HS16207 | 3 | *L.fuscus* | ♂ | liver | 1 | 42.4 | 107.3 | - | - | - | - | - | - |
|  | HS14222 | 3 | *L.fuscus* | ♂ | liver | 1 | 21.3 | 81.9 | + | - | - | - | - | - |
|  | HS14235 | 4 | *L.fuscus* | ♀ | liver | 1 | 33.9 | 99.9 | + | + | - | - | - | - |
|  | HS14237 | 4 | *L.fuscus* | ♀ | liver | 1 | 38.2 | 95.7 | - | - | - | - | - | - |
|  | HS14245 | 4 | *L.fuscus* | ♀ | liver | 1 | 31.5 | 93.5 | - | - | - | - | - | - |
|  | HS14250 | 4 | *L.fuscus* | ♂ | liver | 1 | 25.9 | 82.9 | - | - | - | - | - | - |
|  | HS14256 | 4 | *L.fuscus* | ♂ | liver | 2 | 68.3 | 115.9 | + | + | - | - | - | - |
|  | HS14310 | 4 | *L.fuscus* | ♂ | liver | 2 | 77.2 | 129.7 | + | + | - | - | - | - |
|  | HS14319 | 4 | *L.fuscus* | ♀ | liver | 1 | 46.3 | 111.9 | + | - | - | - | - | - |
|  | HS14329 | 4 | *L.fuscus* | ♀ | liver | 1 | 31.3 | 101.5 | + | + | - | - | - | - |
|  | HS14337 | 4 | *L.fuscus* | ♀ | liver | 1 | 35.6 | 100.2 | + | + | - | - | - | - |
|  | HS14142 | 3 | *M. limnophilus* | ♀ | liver | 1 | 67.9 | 110.5 | + | + | - | - | - | - |
|  | HS14156 | 3 | *M. limnophilus* | ♀ | liver | 1 | 44.5 | 114.8 | + | + | - | - | - | - |
|  | HS14190 | 3 | *M. limnophilus* | ♀ | liver | 1 | 43.1 | 122.8 | + | + | - | - | - | - |
|  | HS14212 | 3 | *M. limnophilus* | ♀ | liver | 1 | 20.8 | 90.2 | + | - | - | - | - | - |
|  | HS14261 | 4 | *M. limnophilus* | ♀ | liver | 1 | 27.9 | 102.5 | - | - | - | - | - |  |
|  | HS14264 | 4 | *M. limnophilus* | ♀ | liver | 1 | 24 | 87.5 | + | + | - | - | - | - |
|  | HS14008 | 1 | *O.curzoniae* | ♀ | liver | 1 | 166.2 | 184 | - | - | - | - | - | - |
|  | HS14012 | 1 | *O.curzoniae* | ♂ | liver | 1 | 162.4 | 180 | - | - | - | - | - | - |
|  | HS14015 | 1 | *O.curzoniae* | ♀ | lung | 1 | 147.8 | 203 | - | - | - | - | - | - |
|  | HS14020 | 1 | *O.curzoniae* | ♀ | liver & lung | 1 | 143.3 | 183 | - | - | - | - | - | - |
|  | HS14021 | 1 | *O.curzoniae* | ♀ | liver | 1 | 116.1 | 161 | - | - | - | - | - | - |
|  | HS14023 | 1 | *O.curzoniae* | ♂ | lung | 1 | 165.7 | 178 | - | - | - | - | - | - |
|  | HS14030 | 1 | *O.curzoniae* | ♂ | liver | 1 | 181.6 | 191 | - | - | - | - | - | - |
|  | HS14033 | 1 | *O.curzoniae* | ♀ | liver | 1 | 132.5 | 158 | - | - | - | - | - | - |
|  | HS14042 | 1 | *O.curzoniae* | ♀ | liver | 1 | 143.5 | 185 | - | - | - | - | - | - |
|  | HS14043 | 1 | *O.curzoniae* | ♀ | liver | 1 | 140.2 | 190 | - | - | - | - | - | - |
|  | HS14056 | 1 | *O.curzoniae* | ♀ | liver | 1 | 131.9 | 177 | - | - | - | - | - | - |
|  | HS14059 | 1 | *O.curzoniae* | ♀ | liver | 1 | 138.5 | 183 | - | - | - | - | - | - |
|  | HS14062 | 1 | *O.curzoniae* | ♀ | liver | 1 | 147.1 | 166 | - | - | - | - | - | - |
|  | HS14063 | 1 | *O.curzoniae* | ♂ | liver | 1 | 162.3 | 176 | - | - | - | - | - | - |
|  | HS14066 | 1 | *O.curzoniae* | ♀ | lung | 1 | 142.5 | 193 | - | - | - | - | - | - |
|  | HS14068 | 2 | *O.curzoniae* | ♀ | liver & lung | 1 | 126.5 | 170 | - | - | - | - | - | - |
|  | HS14072 | 2 | *O.curzoniae* | ♂ | liver | 1 | 160.8 | 180 | - | - | - | - | - | - |
|  | HS14074 | 2 | *O.curzoniae* | ♂ | liver | 1 | 148.6 | 161 | - | - | - | - | - | - |
|  | HS14077 | 2 | *O.curzoniae* | ♀ | liver | 1 | 135.6 | 180 | - | - | - | - | - | - |
|  | HS14078 | 2 | *O.curzoniae* | ♀ | lung | 1 | 145.4 | 190 | - | - | - | - | - | - |
|  | HS14081 | 2 | *O.curzoniae* | ♂ | liver & lung | 1 | 150.6 | 185 | - | - | - | - | - | - |
|  | HS14083 | 2 | *O.curzoniae* | ♂ | liver | 1 | 167.9 | 180 | - | - | - | - | - | - |
|  | HS14084 | 2 | *O.curzoniae* | ♀ | liver | 1 | 139.6 | 182 | - | - | - | - | - | - |
|  | HS14085 | 2 | *O.curzoniae* | ♀ | liver | 1 | 146.5 | 190 | - | - | - | - | - | - |
|  | HS14093 | 2 | *O.curzoniae* | ♂ | liver | 1 | 154.5 | 190 | - | - | - | - | - | - |
|  | HS14095 | 2 | *O.curzoniae* | ♀ | liver | 1 | 162.3 | 183 | - | - | - | - | - | - |
|  | HS14096 | 2 | *O.curzoniae* | ♂ | liver | 1 | 179.5 | 197 | - | - | - | - | - | - |
|  | HS14107 | 2 | *O.curzoniae* | ♂ | liver | 1 | 177.7 | 185 | - | - | - | - | - | - |
|  | HS14108 | 2 | *O.curzoniae* | ♂ | liver | 1 | 108.7 | 170 | - | - | - | - | - | - |
|  | HS14110 | 2 | *O.curzoniae* | ♀ | liver & lung | 1 | － | － | - | - | - | - | - | - |
|  | HS14113 | 2 | *O.curzoniae* | ♀ | liver | 1 | － | － | - | - | - | - | - | - |
|  | HS14116 | 2 | *O.curzoniae* | ♂ | liver | 1 | － | － | - | - | - | - | - | - |
|  | HS14119 | 2 | *O.curzoniae* | ♂ | liver | 1 | － | － | - | - | - | - | - | - |
|  | HS14120 | 2 | *O.curzoniae* | ♀ | liver | 1 | － | － | - | - | - | - | - | - |
|  | HS14126 | 2 | *O.curzoniae* | ♀ | liver & lung | 2 | － | － | - | - | + | + | - | - |
|  | HS14130 | 2 | *O.curzoniae* | ♀ | lung | 1 | － | － | + | - | - | - | - | - |
|  | HS14131 | 2 | *O.curzoniae* | ♀ | liver | 1 | － | － | - | - | - | - | - | - |
| Liver samples^c^ | HS14009 | 1 | *C.longicaudatus* | ♀ | - | 0 | 46.1 | 102.1 | - | - | - | + | - | - |
|  | HS14145 | 2 | *L.fuscus* | ♀ | - | 0 | 67.9 | 118.6 | - | - | + | + | - | - |
|  | HS14175 | 3 | *L.fuscus* | ♂ | - | 0 | 49.5 | 125.1 | - | - | - | + | - | - |
|  | HS14279 | 4 | *L.fuscus* | ♀ | - | 0 | 38.2 | 111.1 | + | - | - | - | - | - |
|  | HS14140 | 2 | *M. limnophilus* | ♂ | - | 0 | 36.1 | 106.1 | - | - | - | + | - | - |
|  | HS14155 | 2 | *M. limnophilus* | ♀ | - | 0 | 40.4 | 110.3 | - | - | + | + | - | - |
|  | HS14165 | 3 | *M. limnophilus* | ♀ | - | 0 | 37.5 | 112.2 | - | - | + | + | - | - |
|  | HS14225 | 4 | *M. limnophilus* | ♀ | - | 0 | 21.1 | 94.6 | - | - | + | + | - | - |
|  | HS14255 | 4 | *M. limnophilus* | ♀ | - | 0 | 19.6 | 80.8 | - | - | - | + | - | - |
|  | HS14016 | 1 | *O.curzoniae* | ♀ | - | 0 | 156.6 | 201 | - | - | - | + | - | - |
|  | HS14045 | 1 | *O.curzoniae* | ♂ | - | 0 | 158 | 175 | - | - | - | + | - | - |
|  | HS14055 | 1 | *O.curzoniae* | ♂ | - | 0 | 166.3 | 184 | - | - | - | + | - | - |
|  | HS14105 | 2 | *O.curzoniae* | ♂ | - | 0 | 133.7 | 186 | - | - | - | + | - | - |
|  | HS14125 | 2 | *O.curzoniae* | ♂ | - | 0 | － | － | + | - | - | - | - | - |
|  | HS14154 | 2 | *P.leucurus* | ♀ | - | 0 | 28.6 | 96.1 | - | - | - | + | - | - |
| Total |  |  |  |  |  |  |  |  | 23 | 15 | 5 | 14 |  |  |

1. Lesion samples were suspect samples collected from small mammals with lesions found in their inner organs when dissecting.
2. Lesion visibility valued the visible lesion size degrees: 0, no visible susceptible lesions; 1, atypical lesions; 2, typical lesions.
3. Liver samples were collected from individuals without visible lesions of *Echinococcus*.

- No data available. Bodies of eight *O. curzoniae* individuals were incomplete when collected, thus no measuring was conducted.

# References

1. Gandon S. Evolution of multihost parasites. Evolution. 2004;58:455–69.
2. Konyaev SV, Yanagida T, Nakao M, Ingovatova GM, Shoykhet YN, Bondarev AL, et al. Genetic diversity of *Echinococcus* spp. in Russia. Parasitology. 2013;140:1637–47.
3. Nakao M, Xiao N, Okamoto M, Yanagida T, Sako Y, Ito A. Geographic pattern of genetic variation in the fox tapeworm *Echinococcus multilocularis*. Parasitol Int. 2009;58:384–9.
4. Geay KM, Jenkins EJ. Introduced and native haplotypes of *Echinococcus multilocularis* in wildlife in Saskatchewan, Canada. J Wildlife Dis. 2015;51:743–8.
5. Laurimaa L, SLld K, Moks E, Valdmann H, Umhang G, Knapp J, et al. First report of the zoonotic tapeworm *Echinococcus multilocularis* in raccoon dogs in Estonia, and comparisons with other countries in Europe. Vet Parasitol. 2015;212:200–5.
6. Yamasaki H, Nakao M, Nakaya K, Schantz PM, Ito A. Genetic analysis of *Echinococcus multilocularis* originating from a patient with alveolar echinococcosis occurring in Minnesota in 1977. Am J Trop Med Hyg. 2008;79:245–7.
